# *SOX4* as biomarker in hepatitis B virus-associated hepatocellular carcinoma

**DOI:** 10.7150/jca.46579

**Published:** 2021-04-19

**Authors:** Jian-Lv Huang, Xiang-Kun Wang, Xi-Wen Liao, Chuang-Ye Han, Ting-Dong Yu, Ke-Tuan Huang, Cheng-Kun Yang, Xiao-Guang Liu, Long Yu, Guang-Zhi Zhu, Hao Su, Wei Qin, Quan-Fa Han, Zheng-Qian Liu, Xin Zhou, Jun-Qi Liu, Xin-Ping Ye, Tao Peng

**Affiliations:** 1 Department of Hepatobiliary Surgery, The First Affiliated Hospital of Guangxi Medical University, Nanning, 530021, Guangxi Zhuang Autonomous Region, People's Republic of China.; 2 Department of Hepatobiliary Surgery, Affiliated Hospital of Guangdong Medical University, Zhanjiang, 524001, Guangdong Province, China.; 3 Department of Hepatobiliary and Pancreatic Surgery, The First Affiliated Hospital of Zhengzhou University, Zhengzhou, 450000, Henan Province, China.

**Keywords:** SOX4, hepatocellular carcinoma, hepatitis B virus, mRNA expression

## Abstract

**Background:** Hepatitis B virus infection is associated with liver disease, including cancers. In this study, we assessed the power of sex-determining region Y (SRY)-related high-mobility group (HMG)-box 4(*SOX4*) gene to predict the clinical course of hepatocellular carcinoma (HCC).

**Methods:** To evaluate the differential expression of *SOX4* and its diagnostic and prognostic potential in HCC, we analyzed the GSE14520 dataset. Stratified analysis and joint-effect analysis were done using *SOX4* and clinical factor. We then designed a nomogram for predicting the clinical course of HCC. Differential *SOX4* expression and its correlation with tumor stage as well as its diagnostic and prognostic value were analyzed on the oncomine and GEPIA websites. Gene set enrichment analysis was explored as well as candidate gene ontology and metabolic pathways modulated by in *SOX4* HCC.

**Results:** Our analysis revealed that the level of *SOX4* was significantly upregulated in tumor issue (*P* <0.001). This observation was validated through oncomine dataset and MERAV analysis (all *P* <0.05). Diagnostic receiver operating characteristic (ROC) analysis of *SOX4* suggested it has diagnostic potential in HCC (GSE14520 dataset: *P* <0.001, area under curve (AUC) = 0.782; Oncomine: (Wurmbach dataset) *P* = 0.002, AUC = 0.831 and (Mas dataset) *P* <0.001, AUC = 0.947). In addition, *SOX4* exhibited high correlation with overall survival of HBV-associated HCC (adjusted *P* = 0.004, hazard ratio (HR) (95% confidence interval (CI)) = 2.055 (1.261-3.349) and recurrence-free survival (adjusted *P* = 0.008, HR (95% CI) = 1.721 (1.151-2.574). These observations which were verified by GEPIA analysis for overall survival (*P* = 0.007) and recurrence-free survival (*P*= 0.096). Gene enrichment analysis revealed that affected processes included lymphocyte differentiation, pancreatic endocrine pathways, and insulin signaling pathway. *SOX4* prognostic value was evaluated using nomogram analysis for HCC 1, 3, and 5-year, survival.

**Conclusion:** Differential *SOX4* expression presents an avenue of diagnosing and predicting clinical course of HCC. In HCC, *SOX4* may affect TP53 metabolic processes, lymphocyte differentiation and the insulin signaling pathway.

## Introduction

Cancers affecting liver tissues have been on the rise, making liver cancer the fourth cause of deaths and sixth most prevalent cancer globally 1. Specifically, hepatocellular carcinoma is the most common type of primary liver cancers. Liver cancer is estimated to be the fourth commonest cancer among Chinese males 2. Majority of liver cancers have been associated hepatitis B virus (HBV) infection. While advances in diagnostic and treatment strategies have improved HCC clinical outcomes, its 5-year survival remains low (<15%) 3. Early detection and more effective management of liver cancer is therefore necessary. While some HCC prognostic biomarkers have been recommended, such as α-fetoprotein (AFP) 4 and PIVKA-II 5, HCC survival is still poor. Better understanding of the mechanisms of HCC development and progression, as well as the identification of novel prognostic biomarkers is needed.

Sex-determining region Y (SRY)-related high-mobility group (HMG)-box(*SOX*) genes are evolutionarily conserved and are thought to be regulate in cell fate determination during development 6. During embryogenesis, this family of genes participates in the development of neuronal tissue, nervous system and as well as skeletal tissue 6. *SOX4* comprises three domains - a serine-rich region, a glycine-rich region and an HMG box 7. This gene participates in tumorigenesis and progression. It has also been verified that *SOX4* regulates lymphocyte differentiation and development, and drive endocardial ridge development 8. It is emerging that *SOX4* is markedly upregulated in various human cancers, including breast cancer 9, 10, colorectal cancer 11, gastric cancer 12 and HCC 13, 14. *SOX4* expression has also been associated with prognosis of some cancer types 7, 15. Lack of *SOX4* expression in normal adult liver does not affect normal liver function 16. Nevertheless, the exact role of *SOX4* gene along the clinical course of HCC is yet to be fully uncovered. This study evaluated the prognostic and diagnostic value of *SOX4* in HBV-associated HCC.

## Materials and methods

### Date source

The GSE14520 dataset for *SOX4* expression, and corresponding clinical data on HBV-related HCC were downloaded from the Gene Expression Omnibus (GEO) database (https://www.ncbi.nlm.nih.gov/geo/query/acc.cgi?acc=GSE14520, accessed December 15, 2017) 17, 18. Data on the expression of *SOX4* in tumor vs. non-tumor tissue were downloaded from metabolic gene rapid visualizer (MERAV, http://merav.wi.mit.edu/, accessed December 15, 2017) 19.

### Bioinformatic analysis and *SOX4* diagnostic potential

In order to investigate the biological function and pathways associated with *SOX4*, we performed a gene ontology (GO) term analysis of *SOX4* using the bionetwork gene ontology tool (BinGO) in Cytoscape_version3.4.0. GeneMANIA (http://www.genemania.org/, accessed December 17, 2017) 20, 21 and STRING (https://string-db.org/, accessed December 17, 2017) 22, 23 were used to investigate SOX4 gene-gene and protein-protein interactions, respectively. In order to explore the diagnostic value of the *SOX4*, we used T-test analysis to compare its expression in tumor vs non-tumor tissues in the GSE14520 dataset and then conducted a receiver operating characteristic (ROC) analysis. Diagnostic value was considered statistically significant when *P*<0.05 and area under curve (AUC) >0.7.

### Survival analysis, joint-effect and stratified analysis

For survival analysis, patient data were divided into 2 categories on the basis of median *SOX4* mRNA expression. RFS (recurrence-free survival) and OS (overall survival) were estimated using Cox proportional hazards regression and Kaplan-Meier models. Clinical factors found to be statistically significant were adjusted for survival analysis and joint effects survival analysis for *SOX4*. *SOX4* gene was then combined with AFP for survival analysis. Furthermore, *SOX4* gene expression was subclassified to effectively perform analyses for clinical factors. Next, factors found to be significant were included in multivariate analysis.

### Gene set enrichment analysis (GSEA)

To investigate the prognostic value of *SOX4* in HCC, the difference in biological functions and pathways between high and low *SOX4* expression groups were analyzed using GSEA (http://software.broadinstitute.org/gsea/index.jsp,accessed December 19, 2017) 24, 25. This analysis evaluated the molecular signatures database (MSigDB) of c2 (c2.cp.kegg.v6.1.symbols) and c5 (c5.all.v6.1.symbols). Differences were considered statistically significant is the *P* value <0.05 and false discovery rate <0.25.

### Nomogram construction of *SOX4* and prognosis-related clinical factors

Nomogram analysis was used to 1, 3-, and 5-year OS and RFS. The nomogram was constructed using prognosis-related clinical factors and *SOX4* expression. Different factors and genes had different expression scores.

### Validation cohorts

To validate the diagnostic value of *SOX4*, we analyzed *SOX4* expression in tumor tissue vs tumor-adjacent normal tissue using the Mas 26 and Wurmbach 27 liver datasets on Oncomine (http://www.oncomine.org/, accessed December 21, 2017). Differential *SOX4* expression analysis was also performed using the aforementioned datasets. *SOX4* expression scatter plots, staging verification and prognosis verification were conducted using gene expression profiling interactive analysis (GEPIA, http://gepia.cancer-pku.cn/index.html, accessed December 21, 2017) 28.

### Statistical analysis

Data were analyzed using SPSS version 24.0 (IBM corporation, Armonk, NY, USA) and R 3.6.0. The log-rank *P* and median survival time (MST) were determined using Kaplan-Meier method. The hazard ratio (HR) and 95% confidence interval (CI) were estimated using univariate and multivariate Cox proportional hazards regression models. Differential SOX4 expression between tumor and non-tumor tissue was analyzed by T-test. *P* < 0.05 was considered significant.

## Results

### Differential expressions and diagnostic analysis

**Figure 1:**
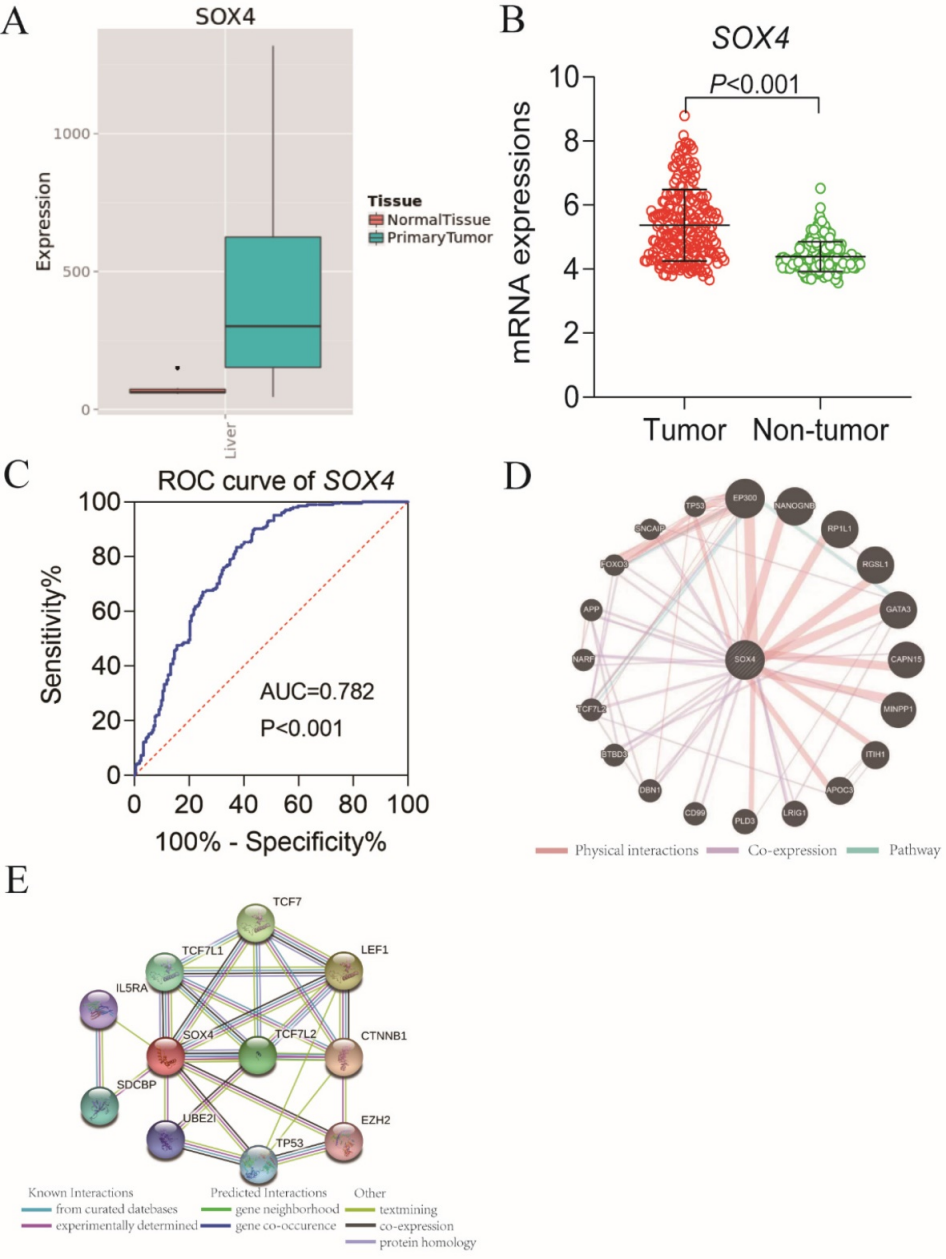
Scatter plots, ROC analysis, and interaction network of *SOX4* gene. (A) Scatter plots of *SOX4* expression by MERAV website. (B) Scatter plots of *SOX4* expression in GSE14520 cohort. (C) Diagnostic receiver operating characteristic curve of *SOX4* in GSE14520 cohort. (D) Gene-gene interaction network of *SOX4* gene. (E) Protein-protein interaction network of *SOX4* protein.

Our analysis of the GSE14520 and MERAV dataset revealed elevated *SOX4* expression in HCC tissues relative to normal tissue (*P <*0.001, Figure 1A-B). The ROC analysis of *SOX4* in the GSE14520 dataset, HBV-related HCC cohort indicated that *SOX4* had a high accuracy of distinguishing tumor tissues from adjacent non-tumor liver tissues (*P* <0.001, AUC of the ROC curves = 0.782; Figure 1C).

### Survival analysis of SOX4 in OS and RFS

In order to avoid the batch effect of microarray data, only the dataset of Affymetrix HT Human Genome U133A Array of GSE14520 was included in the current study. Because most of the patients in GSE14520 were HBV-related HCC, we excluded those patients without HBV infection reports and survival information. As a result, there were 212 HBV-related HCC patients were included in the current study, and all of the 212 HBV-related HCC patients and had prognosis information. Our analysis of suggested that the cirrhosis and Barcelona Clinic Liver Cancer (BCLC) stage significantly correlate with OS and RFS (OS: *P* = 0.041,* <*0.001; RFS: 0.036, *<*0.001 respectively; Supplementary Table 1) 29. Tumor size and APF significantly associate with OS (*P* = 0.002, 0.049, respectively; Supplementary Table 1). This analysis further revealed that gender significantly correlates with RFS (*P* = 0.021; Supplementary Table 1).

**Figure 2:**
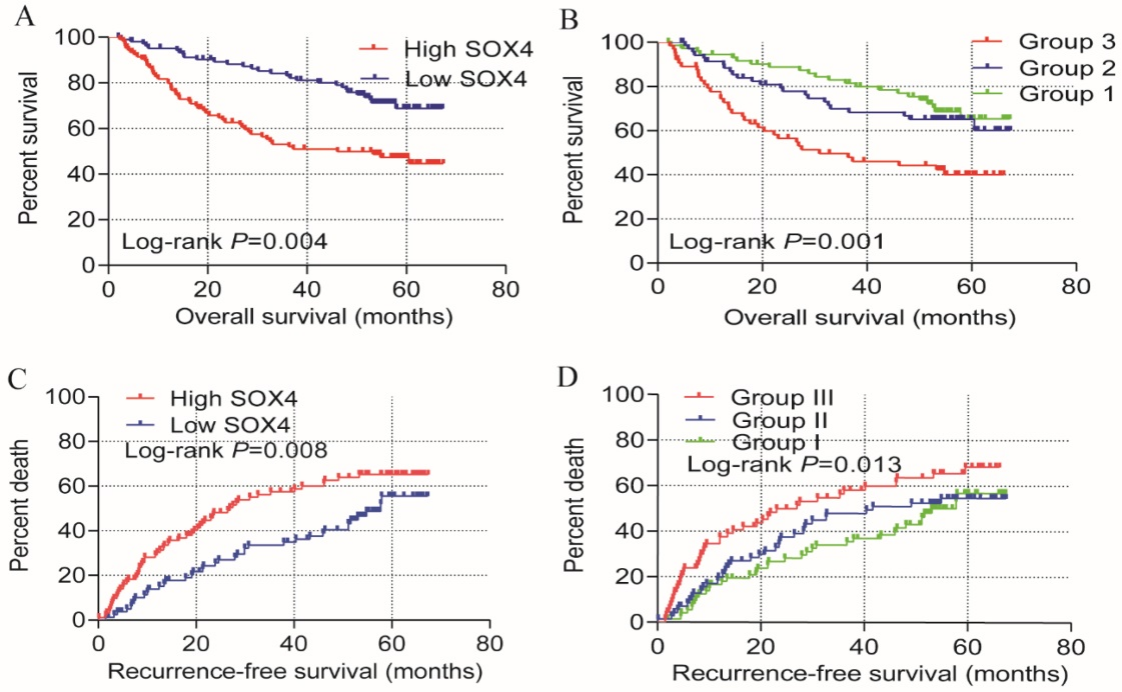
Univariate prognosis analysis and joint-effect analysis of *SOX4*. (A) OS analysis plot of *SOX4*. (B) Joint-effect OS analysis plot of *SOX4* and AFP. (C) RFS analysis plot of *SOX4*. (D) Joint-effect RFS analysis plot of *SOX4* and AFP. OS = overall survival, RFS = recurrence-free survival.

Univariate OS analysis, revealed that *SOX4* expression significantly correlates with survival (crude *P<*0.001, HR = 2.397, 95% CI = 1.522-3.775; Table 1, Figure 2). Similar results were obtained from multivariate OS analysis (adjusted *P* = 0.004, HR = 2.055, 95% CI = 1.261-3.349; Table 1, Figure 2). Univariate analysis of RFS revealed that *SOX4* expression significantly correlates with survival (crude *P* = 0.001, HR = 1.896, 95% CI = 1.307-2.750; Table 1, Figure 2) and similar results were obtained by multivariate RFS analysis (adjusted *P* = 0.008, HR = 1.721, 95% CI=1.151-2.574; Table 1, Figure 2).

### Stratified analysis and joint-effect analysis

**Figure 3:**
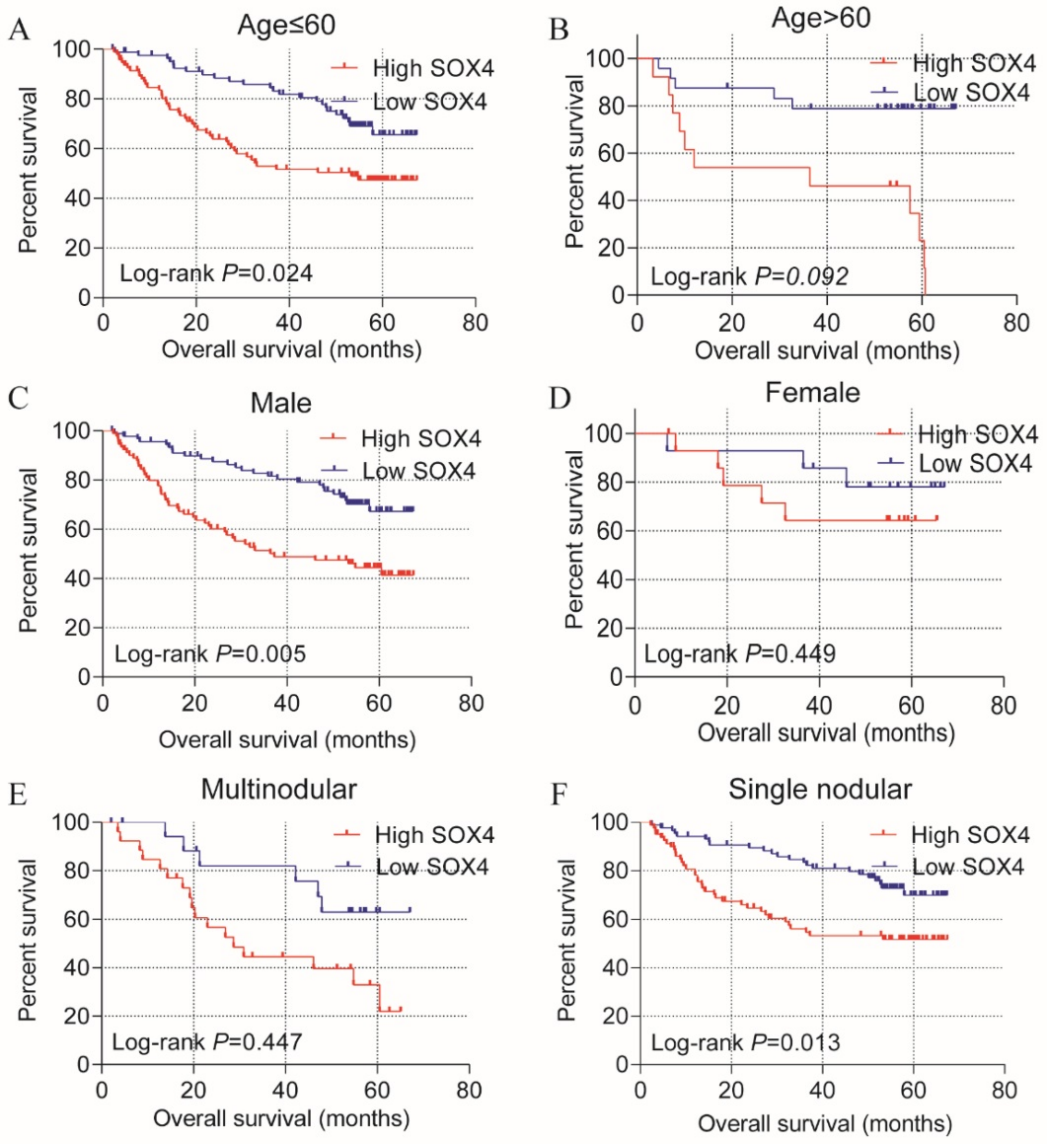
Statistical OS analysis plot of *SOX4* by clinical factors. (A-B): Statistical OS analysis plot of *SOX4* by, (A) age ≤60, (B) >60, (C) male, (D) female, (E) multinodular, and (F) single nodular.

**Figure 4:**
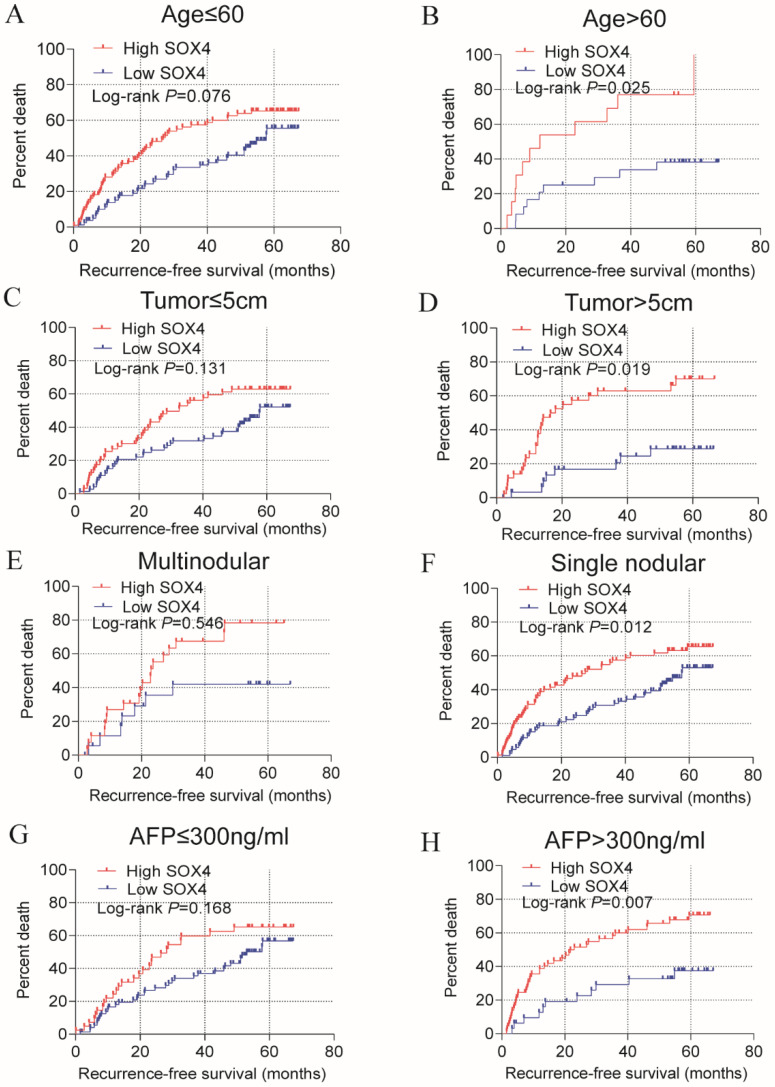
Statistical RFS analysis plot of *SOX4* by clinical factors. (A-B) Statistical OS analysis plot of *SOX4* by (A) age ≤60, (B) >60, (C) tumor ≤5 cm, (D) tumor >5 cm, (E) multinodular, (F) single nodular, (G) AFP ≤300 ng/ml and (I) AFP >300 ng/ml.

Stratified analysis of how *SOX4* influences OS and RFS indicated age (≤60), being male and single nodular significantly correlate with HCC OS (*P* = 0.024, 0.005 and 0.013, respectively; Figure 3; Table 2). An age of >60 years, tumor size >5 cm, single nodular and AFP >300 ng/mL were associated with a longer RFS relative to others (*P* = 0.025, 0.019, 0.012 and 0.007 respectively; Figure 4; Table 2).

Analysis of survival on the GSE14520 cohort revealed that *SOX4* expression is significantly associated with HCC OS and RFS. Previous studies have reported that AFP is associated with the HCC diagnosis and prognosis. We therefore investigated the combined role of *SOX4* and AFP expression on HCC OS and RFS. Analysis of the GSE14520 cohort indicated that the risk of death and recurrence was significantly higher in patients exhibiting high AFP and *SOX4* expression when compared to those with low (Figure 2; Table 3).

### Prognostic nomogram for survival prediction

Next, we constructed a nomogram for OS based on the following clinical features: BCLC stage, cirrhosis, serum AFP level, tumor size and *SOX4* expression. The following clinical features were used to construct a nomogram for RFS: BCLC stage, cirrhosis, gender and *SOX4* expression. The nomograms may enable individualized prognosis prediction. Nomogram analysis was performed for the probabilities of 1-, 3-and 5-year OS (Figure 7) and RFS (Figure 8). These analyses revealed that *SOX4* expression levels were correlated with the patients' clinical prognosis.

### Bioinformatics analysis of *SOX4* gene

**Figure 5:**
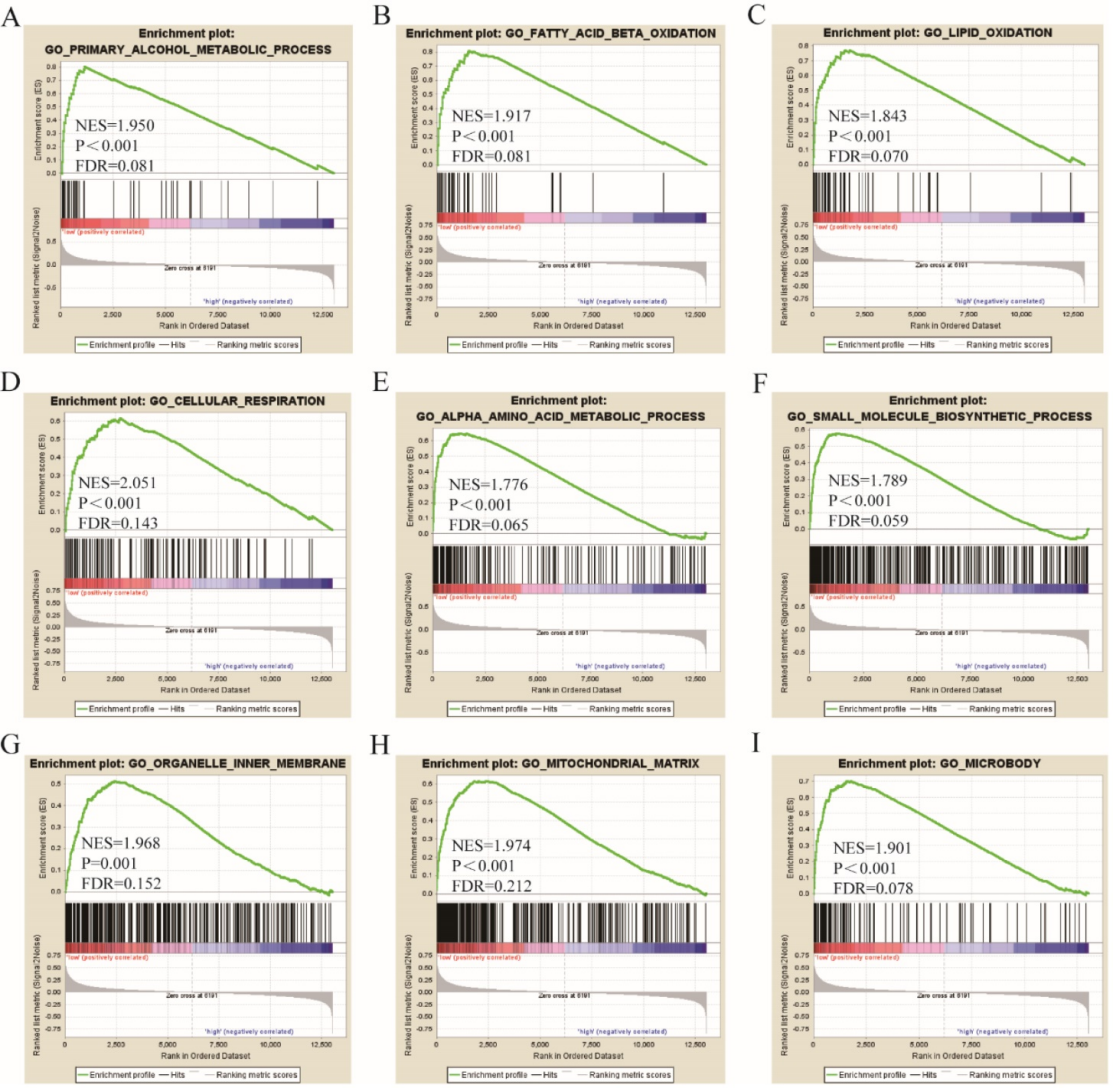
Gene ontology analysis results of *SOX4* gene.

**Figure 6:**
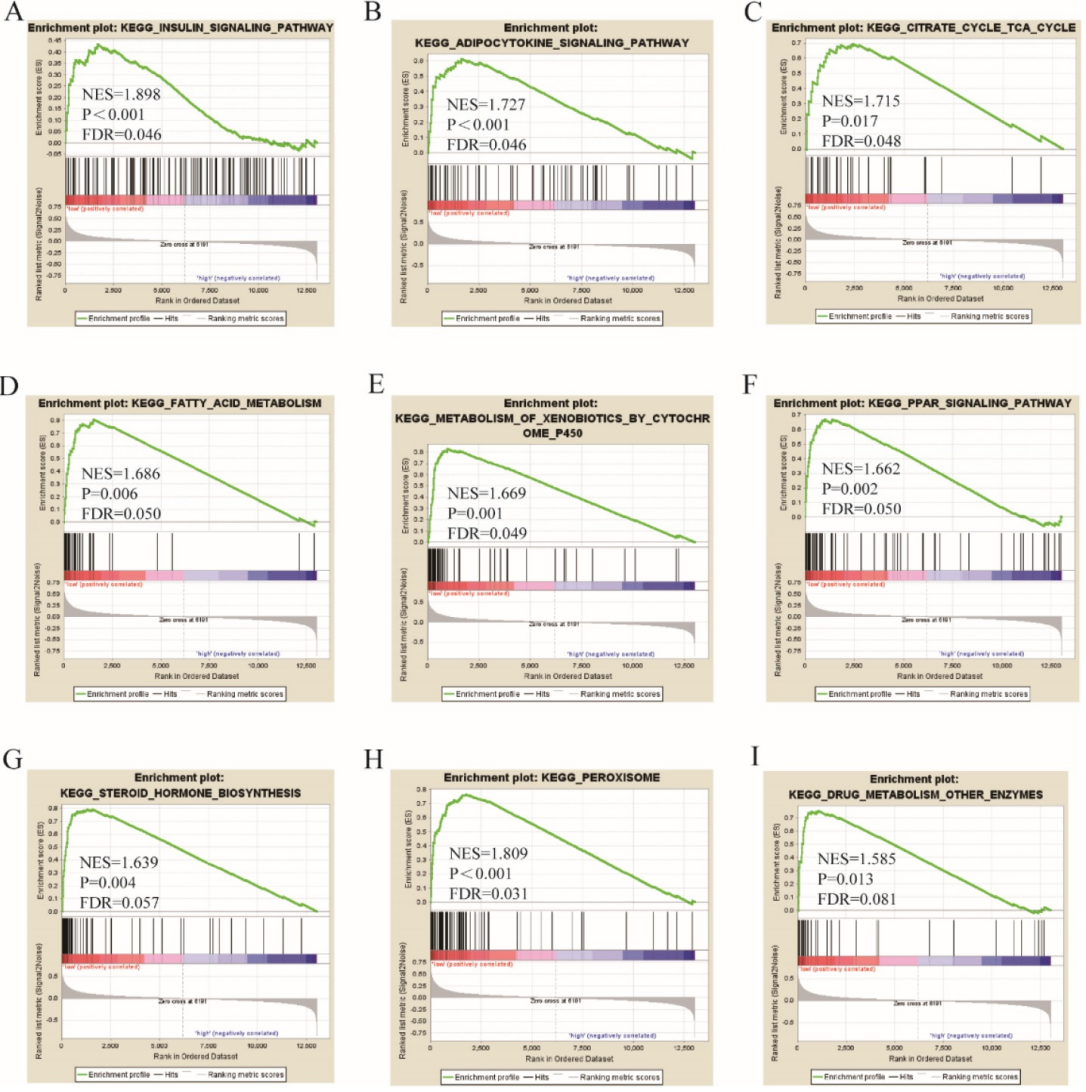
KEGG pathway analysis results of *SOX4* gene.

**Figure 7:**
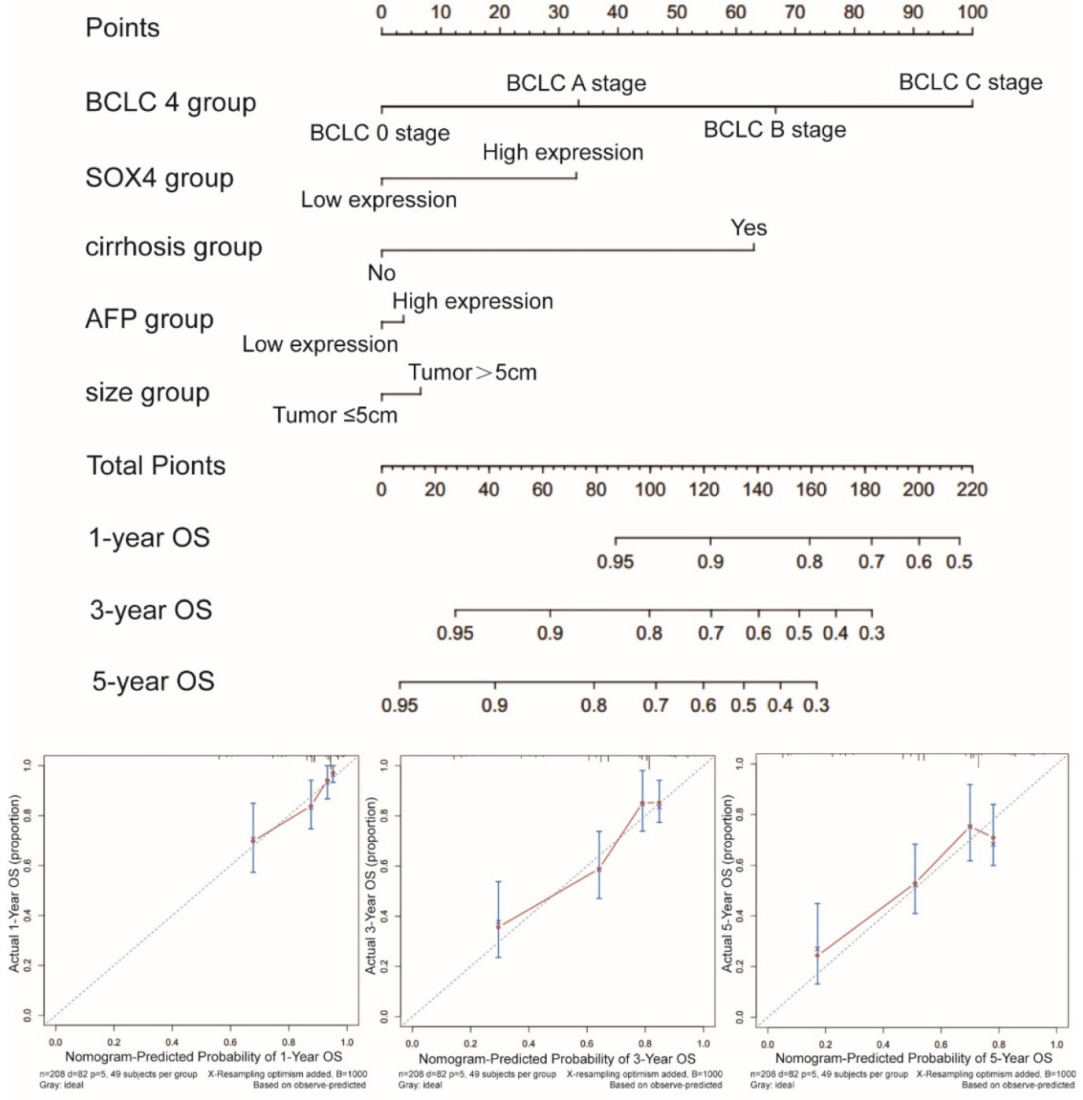
Nomograms constructed using OS-related clinical factors and *SOX4*.

**Figure 8:**
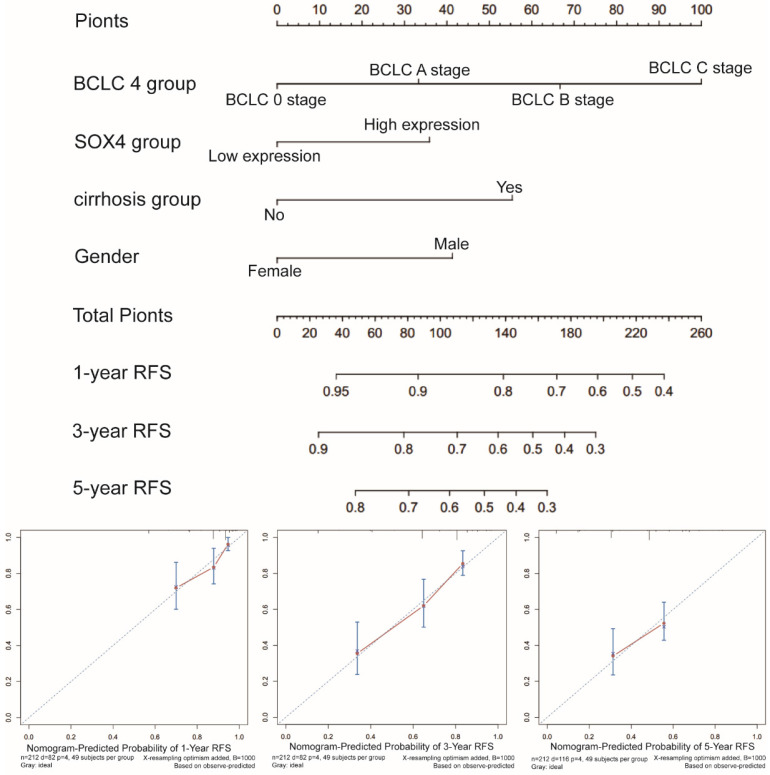
Nomograms constructed using RFS-related clinical factors and *SOX4*.

Go term analysis indicated that *SOX4* gene is involved in the modulation primary alcohol metabolic processes, fatty acid beta oxidation, lipid oxidation, cellular respiration, alpha amino acid metabolic process, small molecule biosynthetic process, organelle inner membrane, mitochondrial matrix and microbody (Figure 5). KEGG functional analysis indicated that the *SOX4* gene is involved in various signaling pathways, including insulin and adipocytokine signaling etc. (Figure 6). Detailed representations of the GSEA results are shown in Figure 5 and 6. The visualized interactions of GO terms were constructed using BinGO (Figure 11). This analysis revealed that *SOX4* may be involved WNT signaling, lymphocyte differentiation and pancreatic endocrine development. Analysis of gene-gene interaction found that *SOX4* is associated with TP53 etc. (Figure 1D) while analysis of protein-protein interaction found that *SOX4* is associated with CTNNB1 and TP53 etc. (Figure 1E).

### Analysis of correlation between SOX4 expression and tumor stage

**Figure 9:**
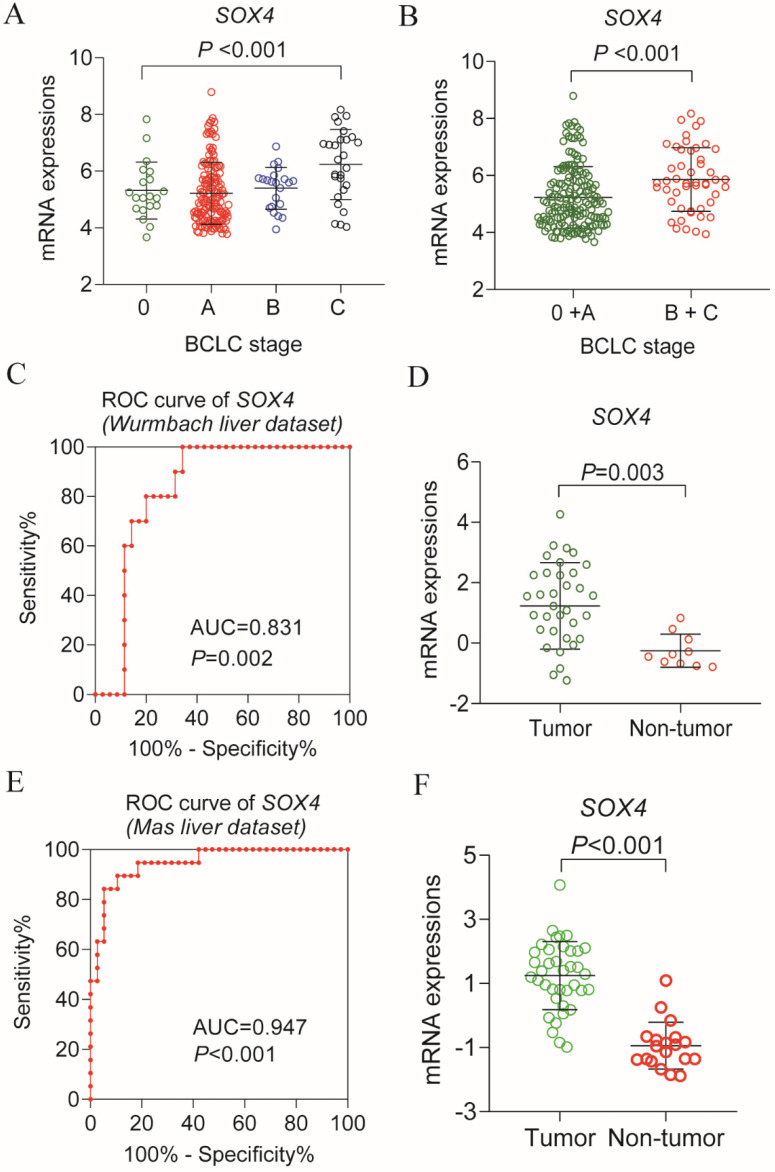
Scatter plot of *SOX4* gene in BCLC stage of GSE14520 cohort and validation using Oncomine database. (A) Scatter plot of *SOX4* gene by BCLC stage (0, A, B, C) of GSE14520 cohort. (B) Scatter plot of *SOX4* gene by BCLC stage (0+A (early stage), B+ C (advance stage) of GSE14520 cohort. (C) ROC curve of *SOX4* in Wurmbach liver dataset. (D) Relative mRNA expressions of *SOX4* in tumor tissue and non-tumor tissue in Wurmbach liver dataset. (E) ROC curve of *SOX4* in Mas liver dataset. (F) Relative mRNA expressions of *SOX4* in tumor tissue and non-tumor tissue in Mas liver dataset.

**Figure 10:**
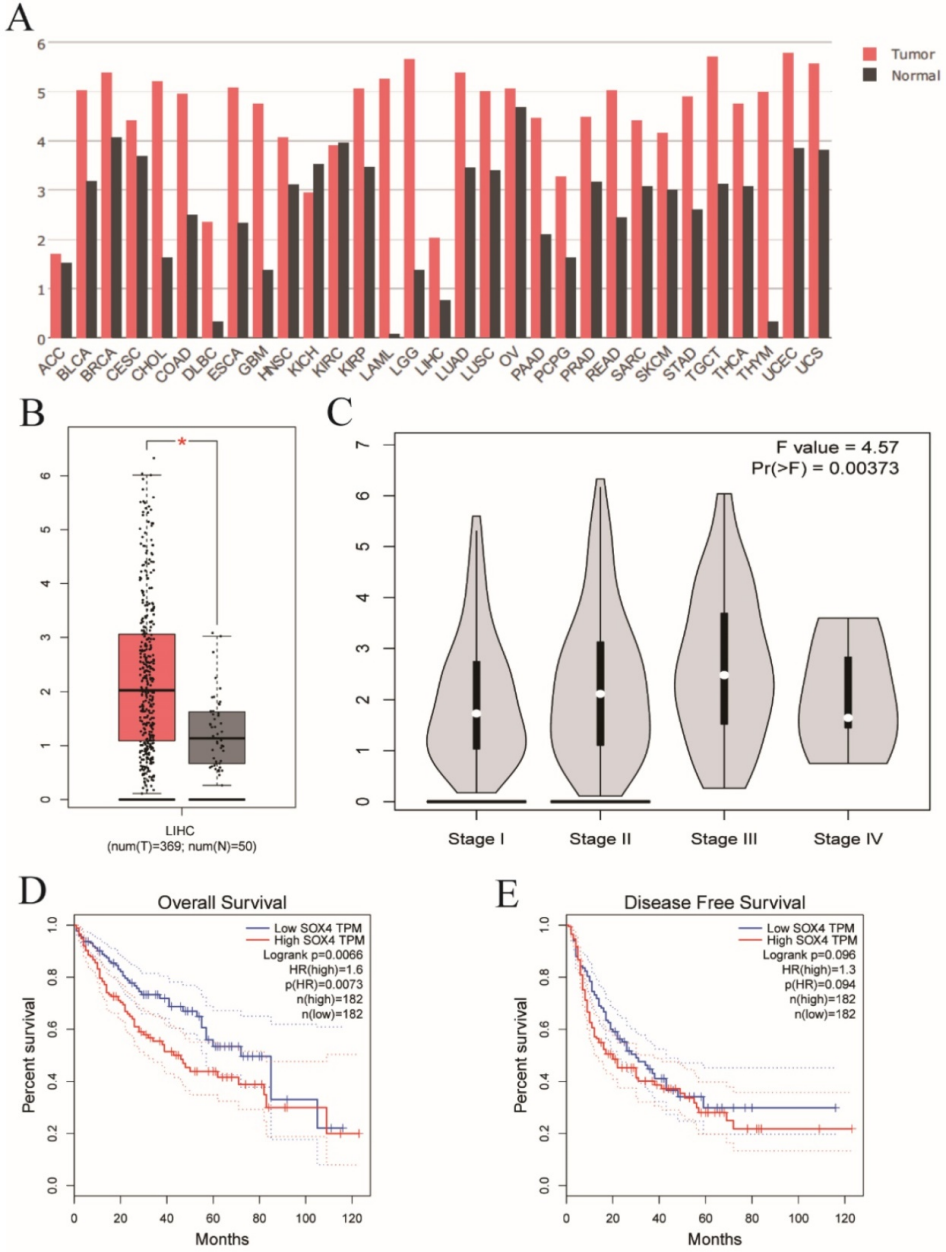
Validation of *SOX4* gene in GEPIA website. (A) The *SOX4* expression profile across all tumor and normal tissues. (B) Differential expressions of *SOX4* in GEPIA website. (C) Violin plot of *SOX4* by tumor stage in GEPIA website. (D) OS analysis plot of *SOX4* in GEPIA website. (E) RFS analysis plot of *SOX4* in GEPIA website.

**Figure 11:**
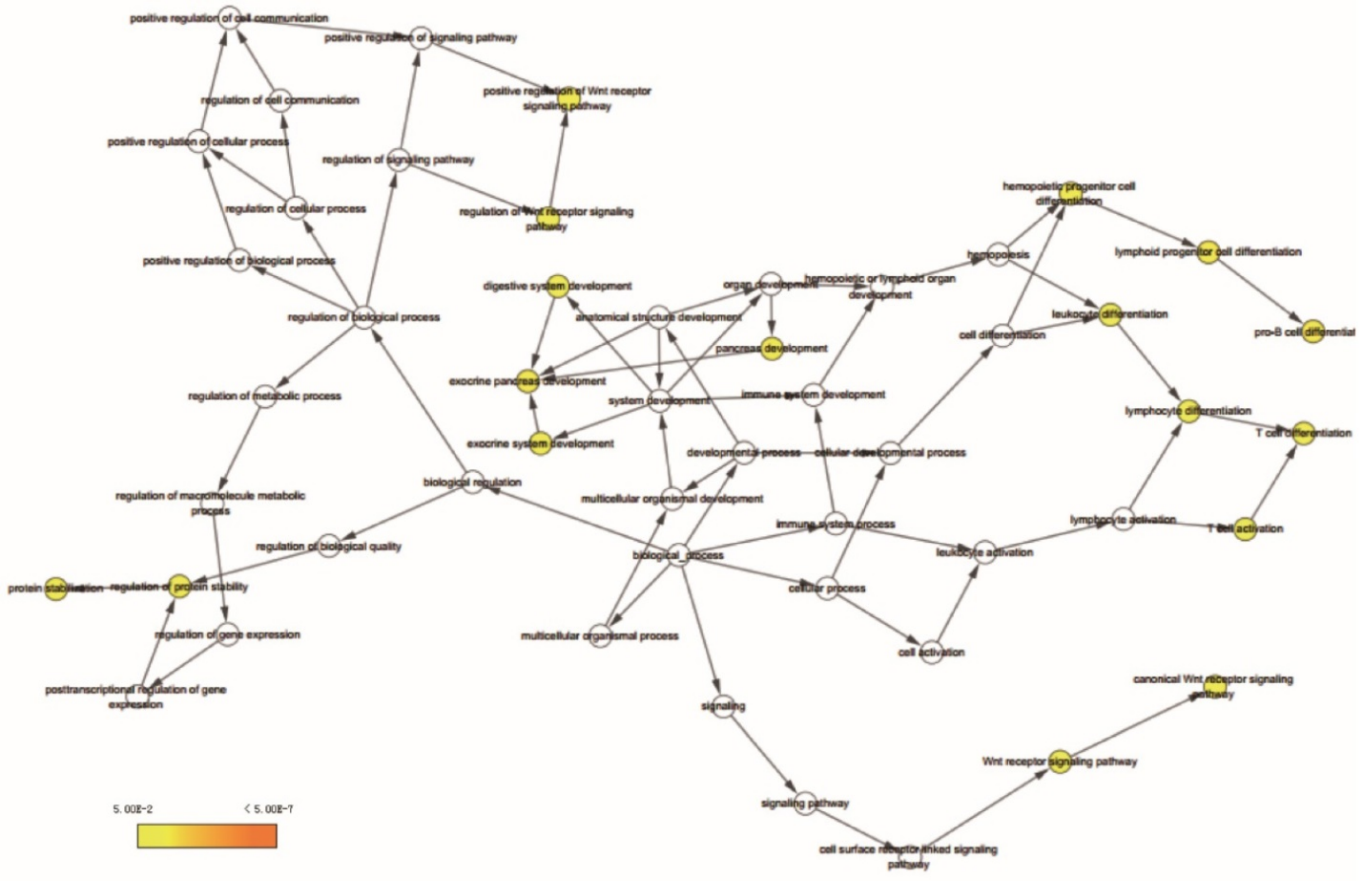
BiNGO analysis results for *SOX4* gene.

Analysis of the GSE14520 dataset for *SOX4* expression at various BCLC stages revealed significantly elevated expression in each BCLC stage *P* <0.001, Figure 9A), but least expressed in the BCLC stage C. Next, we combined BCLC stage 0 and A to constitute the early-stage cancer category and BCLC stage B and C to constitute the advanced-stage cancer category. Interestingly, there was significance lower in former one. Similar results were obtained by GEPIA analysis (*P* = 0.00373; Figure 10C).

### Differential expression, diagnostic and prognostic validation analysis

Next, we analyzed *SOX4* expression in the Wurmbach and Mas liver datasets and found markedly elevated *SOX4* mRNA levels in tumor tissue in relative to normal tissue (*P* = 0.003, <0.001, respectively; Figure 9D, F). Moreover, the potential diagnostic value of SOX4 expression was revealed by ROC analysis of these two databases (AUC = 0.831, 0.947 respectively; *P* = 0.002, <0.001, respectively; Figure 9C, E). *SOX4* expression was also found to be significantly upregulated in tumor tissue following GEPIA analysis (Figure 10A and B). Analysis of the possible impact of *SOX4* expression on survival indicated that patients with low *SOX4* expression levels in the GEPIA analysis, exhibit longer OS relative to those with high expression (*P* = 0.007, Figure 10D). Similar results were obtained for RFS (*P* = 0.096, Figure 10E), although this was not statistically significant. In addition, differences in *SOX4* gene at various stages of HCC were statistically significant (*P* = 0.004; Figure 10C).

## Discussion

Here, we assessed the relationship between *SOX4* levels and various parameters of HBV-related HCC. Results reveal that *SOX4* gene possesses significant value for HCC diagnosis, a finding that is in agreement with previous reports (Wurmbach E et al. and Mas VR et al.) 26, 27. In addition, we find that low SOX4 expression correlates with better HCC prognosis. Next, we carried out joint-effect and stratified analyses of the value of SOX4 as a prognostic indicator in HCC. GSEA analysis indicated that *SOX4* positively modulates primary alcohol metabolic process, fatty acid beta oxidation, lipid oxidation, cellular respiration, small molecule biosynthetic process, alpha amino acid metabolic process, organelle inner membrane, mitochondrial matrix and microbody.

The *SOX4* gene belongs to group C *SOX* transcription factors 30. The products of these genes consist of three domains: a serine-rich region (SRR, aa 333-397), which encodes a protein of 474 amino acids (aa), a glycine-rich region (aa 152-227) and an HMG box (aa 57-135) 30, 31. The HMG box possessed DNA binding, which has been take part in various developmental processes through its transcriptional activity, while SRR domain acts as a deactivation domain. Glycine-rich central region (CD), located between the SRR region and the HMG box is a recently identified functional region that promotes apoptosis 15, 31.

The *SOX4* gene modulates tumor development and growth, epithelial-mesenchymal transition and metastasis 14, 32-34. Furthermore, *SOX4* drives several components of the RNAi machinery, transcriptional regulators, and cellular proteins 35-37. Thus, *SOX4* is a momentous transcription factor that regulates various cellular functions.

Multiple studies have reported the action of *SOX4* as an oncogene in solid tumors 7, 38, 39. It has been reported that *SOX4* is upregulated in various malignancies, including HCC, pancreatic cancer, bladder carcinoma, prostate cancer, breast cancer, colorectal cancer, gastric cancer and melanoma 11, 12, 14, 32, 40-44, raising the potential of this gene as a diagnostic marker. The Mas and Wurmbch liver cancer datasets have reported that *SOX4* is highly expressed in hepatitis C virus-associated HCC 26, 27, which is consistent with our results. Moreover, various reports suggest that *SOX4* can aid in predicting marker in some cancer types. High *SOX4* expression has been associated with poor prognosis in prostate cancer, gastric cancer, colorectal cancer, breast cancer and HCC 11, 12, 14, 32, 45. On the contrary, low expression has been associated with better prognosis in bladder carcinoma and melanoma 41, 43.

Majority of HCCs are attributable to HBV infection 46, 47. Shang et al. reported that HBV increases expression of *SOX4* gene by upregulating transcription factor YY1 via the mitogen-activated protein kinase pathway, epigenetically suppressing miR-203, miR-335, and miR-129-2 by protecting *SOX4* from HBsAg mediated degradation 48. On the other hand, *SOX4* has been shown to promote HBV replication by stimulating viral DNA replication and protein expression in liver cancer cells 49. As a consequence, *SOX4* interacts with HBV and synergistically promotes the occurrence and development of HCC.

It was initially found that* SOX4* acted as a transcription factor that drive B and T lymphocyte differentiation 8, 50. Wilson *et al*. have reported that *SOX4* is involved in pancreatic endocrine development 49. It has been reported that WNT pathway promotes SOX4 expression in colorectal cancer (Van der Flier LG *et al.* and Reichling T *et al.*) 51, 52. SOX4 in turn enhances WNT pathway by stabilizing beta-catenin and directly promoting transcription factor 4 expression 35, 52-55. Multiple studies have demonstrated that *SOX4* interacts with the tumor suppressor p53 during DNA-damage and apoptosis in HCC 15, 56, 57. A study by Yang Jiao et al. reported that tribbles homolog3 is a *SOX4* target 58. Tribbles homolog3 is a pseudo-kinase that disrupts the insulin signaling pathway in the liver by binding to Protein Kinase B and blocking its activation 59, 60. However, none has verified the link between TP53, insulin signaling and *SOX4*. Based on our findings, we hypothesis that *SOX4* may modulate TP53 activity and insulin signaling pathway. However, the mechanism still needs further investigated. Consistent with the aforementioned studies, our data show that *SOX4* might influence WNT signaling, lymphocyte differentiation and TP53 activity.

Herein, we report that *SOX4* is elevated in HCC with BCLC stage B+C than with BCLC stage 0+A. The OS and RFS nomograms indicated that *SOX4* is associated with HCC prognosis. Previous studies have shown that *SOX4* expression is upregulated in breast cancer 9 and promotes HCC metastases 14, suggesting it might lead to poor metastasis-free survival. It has been reported that *SOX4* contributes to hepatocarcinogenesis and its expression can reflect the clinical course of HCC after surgical resection 15.

This study is limited by the small sample size, consisting of 212 HBV-associated liver cancer cases. Future studies should utilize a larger sample size. Our analysis was limited to HBV-associated HCC. It is necessary to explore the diagnostic and prognostic value of *SOX4* all the HCCs, irrespective of HBV status. Since the data of the two cohorts in this study are from public databases, there is no additional validation cohort. This study still needs to be independently verified in an additional cohort. Relative to past studies, this study only assessed the relationship between *SOX4* RNA levels and HCC clinical course. Thus, further investigation is advocated to provide better understanding.

## Conclusions

This study found that *SOX4* expression is significantly upregulated in HCC tumor tissues. Our data indicate that this gene has potential value in HCC diagnosis. Further survival analysis of *SOX4* gene in two cohorts suggests that it significantly correlates with HCC OS and RFS. Bioinformatics analysis suggested that *SOX4* may affect HCC prognosis by modulating TP53 activity, lymphocyte differentiation, pancreatic endocrine development and insulin signaling.

## Supplementary Material

Supplementary table S1.

## Figures and Tables

**Table 1:** Prognostic analysis of *SOX-4* gene in HBV-related HCC of GSE14250 cohort

Type	Gene expression	Patients (n=212)	OS					
			NO. of event	MRT (months)	Crude HR	Crude *P*	Adjusted HR (95% CI)	Adjusted *P^#^*
OS	Low	106	29	NA	Ref.		Ref.	
	High	106	53	46.1	2.397 (1.522-3.775)	0.000	2.055 (1.261-3.349)	**0.004**
RFS	Low	106	47	57.9	Ref.		Ref.	
	High	106	69	26.4	1.896 (1.307-2.750)	0.001	1.721 (1.151-2.574)	0.008

**Note: #**: P values were adjusted for age, gender, tumor size, multinodular, cirrhosis, AFP and BCLC stage; Bold indicates significant *P* values.

**Table 2:** Stratified analysis of *SOX-4* for overall survival and recurrence-free survival

Variables	Overall survival				Recurrence-free survival			
	Low	High	Adjusted HR (95%CI)	Adjusted *P* value	Low	High	Adjusted HR (95%CI)	Adjusted *P* value
**Age (years)**								
≤60	80	92	1.830 (1.084-3.090)	0.024	80	92	1.469 (0.960-2.246)	0.076
>60	23	13	2.947 (0.839-10.352)	0.092	24	13	2.890 (1.114-7.297)	0.025
**Gender**								
Male	90	90	2.069 (1.251-3.420)	0.005	92	91	1.617 (1.085-2.409)	0.018
Female	13	15	2.068 (0.315-13.578)	0.449	14	15	1.744 (0.352-8.643)	0.496
**Tumor size (cm)**								
≤5	73	63	1.322 (0.684-2.591)	0.399	74	63	1.450 (0.895-2.349)	0.131
>5	30	42	3.413 (1.523-7.646)	0.003	31	43	2.237 (1.143-4.377)	0.019
**Cirrhosis**								
Yes	93	98	1.164 (0.695-1.949)	0.563	96	99	1.636 (1.103-2.425)	0.014
No	10	7	2.121 (0.127-35.360)	0.600	10	7	0.878 (0.126-6.136)	0.896
**Multinodular**								
Yes	19	26	1.489 (0.534-4.153)	0.447	19	26	1.322 (0.535-3.268)	0.546
No	84	79	2.044 (1.163-3.592)	0.013	87	80	1.725 (1.130-2.636)	0.012
**AFP (ng/ml)**								
≤300	74	41	1.539 (0.791-2.992)	0.204	74	41	1.448 (0.856-2.451)	0.168
>300	29	64	3.553 (1.542-18.186)	0.003	30	64	2.557 (1.297-5.039)	0.007
**BCLC stage**								
0	11	9	1.871 (0.117-29.956)	0.658	11	9	4.624 (0.686-31.164)	0.116
A	78	61	1.773 (0.969-3.245)	0.063	81	62	1.399 (0.884-2.216)	0.152
B	8	14	1.206 (0.247-5.898)	1.206	8	14	1.026 (0.310-3.393)	0.996
C	6	21	3.801 (0.838-17.234)	3.801	6	21	2.170 (0.630-7.471)	0.220

**Table 3:** Joint-effect analysis of SOX4 and AFP for overall survival and recurrence-free survival

Type	Group	SOX4 expression	APF expression	No. of event	MST (month)	Crude HR (95%CI)	Crude *P* value	Adjusted HR (95%CI)	Adjusted* P* value^#^
OS	1	Low	Low	22/74	55.6	Ref.	0.001	Ref.	0.009
	2	Low	High	24/71	51.0	1.238 (0.694-2.208)	0.470	1.380 (0.718-2.651)	0.333
		High	Low						
	3	High	High	36/64	37.6	2.617 (1.537-4.455)	<0.001	4.667 (1.631-13.358)	0.004
RFS	1	Low	Low	36/74	45.5	Ref.	0.013	Ref.	0.163
	2	Low	High	37/71	41.3	1.136 (0.718-1.797)	0.587	0.960 (0.598-1.542)	0.867
		High	Low						
	3	High	High	43/64	31.4	1.861 (1.194-2.901)	0.006	1.537 (0.964-2.452)	0.071

**Note:** Group 1 stands for a combination of AFP low expression and SOX4 low expression; Group 2 stands for combinations of AFP low expression and SOX4 high expression, as well as AFP high expression and SOX4 low expression; Group 3 stand for a combination of AFP high expression and SOX4 high expression.

## References

[1] Feng RM, Zong YN, Cao SM, Xu RH (2019). Current cancer situation in China: good or bad news from the 2018 Global Cancer Statistics?. Cancer communications.

[2] Cai Z, Liu Q (2019). Understanding the Global Cancer Statistics 2018: implications for cancer control. Science China Life sciences.

[3] DiStefano JK, Davis B (2019). Diagnostic and Prognostic Potential of AKR1B10 in Human Hepatocellular Carcinoma. Cancers.

[4] Zhao YJ, Ju Q, Li GC (2013). Tumor markers for hepatocellular carcinoma. Molecular and clinical oncology.

[5] Qi F, Zhou A, Yan L, Yuan X, Wang D, Chang R The diagnostic value of PIVKA-II, AFP, AFP-L3, CEA, and their combinations in primary and metastatic hepatocellular carcinoma. Journal of clinical laboratory analysis. 2019: e23158.

[6] Pevny LH, Lovell-Badge R (1997). Sox genes find their feet. Current opinion in genetics & development.

[7] Vervoort SJ, van Boxtel R, Coffer PJ (2013). The role of SRY-related HMG box transcription factor 4 (SOX4) in tumorigenesis and metastasis: friend or foe?. Oncogene.

[8] Schilham MW, Clevers H (1998). HMG box containing transcription factors in lymphocyte differentiation. Seminars in immunology.

[9] Tavazoie SF, Alarcon C, Oskarsson T, Padua D, Wang Q, Bos PD (2008). Endogenous human microRNAs that suppress breast cancer metastasis. Nature.

[10] Song GD, Sun Y, Shen H, Li W (2015). SOX4 overexpression is a novel biomarker of malignant status and poor prognosis in breast cancer patients. Tumour biology: the journal of the International Society for Oncodevelopmental Biology and Medicine.

[11] Wang B, Li Y, Tan F, Xiao Z (2016). Increased expression of SOX4 is associated with colorectal cancer progression. Tumour biology: the journal of the International Society for Oncodevelopmental Biology and Medicine.

[12] Pang L, Li B, Zheng B, Niu L, Ge L (2017). miR-138 inhibits gastric cancer growth by suppressing SOX4. Oncology reports.

[13] Zheng JH, Jian ZX, Jin HS, Chen SC, Wang GY (2010). [Expression of SOX4 gene and early recurrence of hepatocellular carcinoma: their relationship and the clinical significance]. Nan fang yi ke da xue xue bao = Journal of Southern Medical University.

[14] Liao YL, Sun YM, Chau GY, Chau YP, Lai TC, Wang JL (2008). Identification of SOX4 target genes using phylogenetic footprinting-based prediction from expression microarrays suggests that overexpression of SOX4 potentiates metastasis in hepatocellular carcinoma. Oncogene.

[15] Hur W, Rhim H, Jung CK, Kim JD, Bae SH, Jang JW (2010). SOX4 overexpression regulates the p53-mediated apoptosis in hepatocellular carcinoma: clinical implication and functional analysis *in vitro*. Carcinogenesis.

[16] Hunt SM, Clarke CL (1999). Expression and hormonal regulation of the Sox4 gene in mouse female reproductive tissues. Biology of reproduction.

[17] Roessler S, Long EL, Budhu A, Chen Y, Zhao X, Ji J (2012). Integrative genomic identification of genes on 8p associated with hepatocellular carcinoma progression and patient survival. Gastroenterology.

[18] Zhao X, Parpart S, Takai A, Roessler S, Budhu A, Yu Z (2015). Integrative genomics identifies YY1AP1 as an oncogenic driver in EpCAM(+) AFP(+) hepatocellular carcinoma. Oncogene.

[19] Shaul YD, Yuan B, Thiru P, Nutter-Upham A, McCallum S, Lanzkron C (2016). MERAV: a tool for comparing gene expression across human tissues and cell types. Nucleic acids research.

[20] Warde-Farley D, Donaldson SL, Comes O, Zuberi K, Badrawi R, Chao P (2010). The GeneMANIA prediction server: biological network integration for gene prioritization and predicting gene function. Nucleic acids research.

[21] Mostafavi S, Ray D, Warde-Farley D, Grouios C, Morris Q (2008). GeneMANIA: a real-time multiple association network integration algorithm for predicting gene function. Genome biology.

[22] Franceschini A, Szklarczyk D, Frankild S, Kuhn M, Simonovic M, Roth A (2013). STRING v9.1: protein-protein interaction networks, with increased coverage and integration. Nucleic acids research.

[23] Szklarczyk D, Morris JH, Cook H, Kuhn M, Wyder S, Simonovic M (2017). The STRING database in 2017: quality-controlled protein-protein association networks, made broadly accessible. Nucleic acids research.

[24] Mootha VK, Lindgren CM, Eriksson KF, Subramanian A, Sihag S, Lehar J (2003). PGC-1alpha-responsive genes involved in oxidative phosphorylation are coordinately downregulated in human diabetes. Nature genetics.

[25] Subramanian A, Tamayo P, Mootha VK, Mukherjee S, Ebert BL, Gillette MA (2005). Gene set enrichment analysis: a knowledge-based approach for interpreting genome-wide expression profiles. Proceedings of the National Academy of Sciences of the United States of America.

[26] Mas VR, Maluf DG, Archer KJ, Yanek K, Kong X, Kulik L (2009). Genes involved in viral carcinogenesis and tumor initiation in hepatitis C virus-induced hepatocellular carcinoma. Molecular medicine.

[27] Wurmbach E, Chen YB, Khitrov G, Zhang W, Roayaie S, Schwartz M (2007). Genome-wide molecular profiles of HCV-induced dysplasia and hepatocellular carcinoma. Hepatology.

[28] Tang Z, Li C, Kang B, Gao G, Li C, Zhang Z (2017). GEPIA: a web server for cancer and normal gene expression profiling and interactive analyses. Nucleic acids research.

[29] Liao X, Liu X, Yang C, Wang X, Yu T, Han C (2018). Distinct Diagnostic and Prognostic Values of Minichromosome Maintenance Gene Expression in Patients with Hepatocellular Carcinoma. Journal of Cancer.

[30] Bowles J, Schepers G, Koopman P (2000). Phylogeny of the SOX family of developmental transcription factors based on sequence and structural indicators. Developmental biology.

[31] Farr CJ, Easty DJ, Ragoussis J, Collignon J, Lovell-Badge R, Goodfellow PN (1993). Characterization and mapping of the human SOX4 gene. Mammalian genome: official journal of the International Mammalian Genome Society.

[32] Zhang J, Liang Q, Lei Y, Yao M, Li L, Gao X (2012). SOX4 induces epithelial-mesenchymal transition and contributes to breast cancer progression. Cancer research.

[33] Rhodes DR, Yu J, Shanker K, Deshpande N, Varambally R, Ghosh D (2004). Large-scale meta-analysis of cancer microarray data identifies common transcriptional profiles of neoplastic transformation and progression. Proceedings of the National Academy of Sciences of the United States of America.

[34] Lourenco AR, Coffer PJ (2017). SOX4: Joining the Master Regulators of Epithelial-to-Mesenchymal Transition?. Trends in cancer.

[35] Sinner D, Kordich JJ, Spence JR, Opoka R, Rankin S, Lin SC (2007). Sox17 and Sox4 differentially regulate beta-catenin/T-cell factor activity and proliferation of colon carcinoma cells. Molecular and cellular biology.

[36] Pramoonjago P, Baras AS, Moskaluk CA (2006). Knockdown of Sox4 expression by RNAi induces apoptosis in ACC3 cells. Oncogene.

[37] Beekman JM, Vervoort SJ, Dekkers F, van Vessem ME, Vendelbosch S, Brugulat-Panes A (2012). Syntenin-mediated regulation of Sox4 proteasomal degradation modulates transcriptional output. Oncogene.

[38] Grimm D, Bauer J, Wise P, Kruger M, Simonsen U, Wehland M (2020). The role of SOX family members in solid tumours and metastasis. Seminars in cancer biology.

[39] Chen X, Zhang L, Zhang T, Hao M, Zhang X, Zhang J (2013). Methylation-mediated repression of microRNA 129-2 enhances oncogenic SOX4 expression in HCC. Liver international: official journal of the International Association for the Study of the Liver.

[40] Mavropoulos A, Devos N, Biemar F, Zecchin E, Argenton F, Edlund H (2005). sox4b is a key player of pancreatic alpha cell differentiation in zebrafish. Developmental biology.

[41] Gunes S, Yegin Z, Sullu Y, Buyukalpelli R, Bagci H (2011). SOX4 expression levels in urothelial bladder carcinoma. Pathology, research and practice.

[42] Scharer CD, McCabe CD, Ali-Seyed M, Berger MF, Bulyk ML, Moreno CS (2009). Genome-wide promoter analysis of the SOX4 transcriptional network in prostate cancer cells. Cancer research.

[43] Jafarnejad SM, Wani AA, Martinka M, Li G (2010). Prognostic significance of Sox4 expression in human cutaneous melanoma and its role in cell migration and invasion. The American journal of pathology.

[44] Huang J, Lu D, Xiang T, Wu X, Ge S, Wang Y (2020). MicroRNA-132-3p regulates cell proliferation, apoptosis, migration and invasion of liver cancer by targeting Sox4. Oncology letters.

[45] Liu P, Ramachandran S, Ali Seyed M, Scharer CD, Laycock N, Dalton WB (2006). Sex-determining region Y box 4 is a transforming oncogene in human prostate cancer cells. Cancer research.

[46] Kanda M, Sugimoto H, Kodera Y (2015). Genetic and epigenetic aspects of initiation and progression of hepatocellular carcinoma. World journal of gastroenterology.

[47] Bray F, Ferlay J, Soerjomataram I, Siegel RL, Torre LA, Jemal A (2018). Global cancer statistics 2018: GLOBOCAN estimates of incidence and mortality worldwide for 36 cancers in 185 countries. CA: a cancer journal for clinicians.

[48] Shang J, Zheng Y, Guo X, Mo J, Xie X, Xiong Y (2015). Hepatitis B virus replication and sex-determining region Y box 4 production are tightly controlled by a novel positive feedback mechanism. Scientific reports.

[49] Wilson ME, Yang KY, Kalousova A, Lau J, Kosaka Y, Lynn FC (2005). The HMG box transcription factor Sox4 contributes to the development of the endocrine pancreas. Diabetes.

[50] Schilham MW, Oosterwegel MA, Moerer P, Ya J, de Boer PA, van de Wetering M (1996). Defects in cardiac outflow tract formation and pro-B-lymphocyte expansion in mice lacking Sox-4. Nature.

[51] Van der Flier LG, Sabates-Bellver J, Oving I, Haegebarth A, De Palo M, Anti M (2007). The Intestinal Wnt/TCF Signature. Gastroenterology.

[52] Reichling T, Goss KH, Carson DJ, Holdcraft RW, Ley-Ebert C, Witte D (2005). Transcriptional profiles of intestinal tumors in Apc(Min) mice are unique from those of embryonic intestine and identify novel gene targets dysregulated in human colorectal tumors. Cancer research.

[53] Lee AK, Ahn SG, Yoon JH, Kim SA (2011). Sox4 stimulates ss-catenin activity through induction of CK2. Oncology reports.

[54] Song DH, Sussman DJ, Seldin DC (2000). Endogenous protein kinase CK2 participates in Wnt signaling in mammary epithelial cells. The Journal of biological chemistry.

[55] Saegusa M, Hashimura M, Kuwata T (2012). Sox4 functions as a positive regulator of beta-catenin signaling through upregulation of TCF4 during morular differentiation of endometrial carcinomas. Laboratory investigation; a journal of technical methods and pathology.

[56] Nikolova D, Chalovska-Ivanova V, Genadieva-Dimitrova M, Eftimov A, Jovanovik R, Janevska V (2018). TP53 Mutation in Correlation to Immunohistochemical Expression of P53 Protein in Patients with Hepatocellular Carcinoma. Open access Macedonian journal of medical sciences.

[57] Pan X, Zhao J, Zhang WN, Li HY, Mu R, Zhou T (2009). Induction of SOX4 by DNA damage is critical for p53 stabilization and function. Proceedings of the National Academy of Sciences of the United States of America.

[58] Jiao Y, Zhao J, Zhang Z, Li M, Yu X, Yang Y (2018). SRY-Box Containing Gene 4 Promotes Liver Steatosis by Upregulation of SREBP-1c. Diabetes.

[59] Koo SH, Satoh H, Herzig S, Lee CH, Hedrick S, Kulkarni R (2004). PGC-1 promotes insulin resistance in liver through PPAR-alpha-dependent induction of TRB-3. Nature medicine.

[60] Du K, Herzig S, Kulkarni RN, Montminy M (2003). TRB3: a tribbles homolog that inhibits Akt/PKB activation by insulin in liver. Science.

