# Supplementary material for: SOX4 as biomarker in hepatitis B virus-associated hepatocellular carcinoma
Source: J Cancer. 2021 Apr 19;12(12):3486–500. doi: 10.7150/jca.46579 (PMC8120190; doi:10.7150/jca.46579)
Supplement: Supplementary file 1 — Supplementary table S1. [file jcav12p3486s1.pdf]

Supplementary table 1. Clinical characteristics of HBV-related HCC patients in GSE14520 cohort

Variables	Patients	RFS				OS			
		No. of events	MRT (months)	HR (95% CI)	*P*	No. of events	MST (months)	HR (95% CI)	*P*
Age (years)									
≤60	175	96	45	1		69	NA	1	
> 60	37	20	48	0.974 (0.602-1.578)	0.916	13	NA	0.864 (0.478-1.564)	0.630
Gender									
Female	29	10	NA	1		8	NA	1	
Male	183	106	40	2.413 (1.120-4.100)	0.021	74	NA	1.704 (0.821-3.534)	0.152
Multinodular									
Single	167	90	49	1		59	NA	1	
Multiple	45	26	28	1.216 (0.785-1.883)	0.382	23	47	1.607 (0.992-2.604)	0.054
Tumor size									
≤5cm	137	73	51	1		46	NA	1	
> 5cm	74	43	28	1.409 (0.966-2.056)	0.075	36	53	1.975 (1.274-3.060)	0.002
Miss[a]	1								
Cirrhosis									
NO	17	5	NA	1		2	NA	1	
Yes	195	111	37	2.612 (1.066-6.402)	0.036	80	NA	4.335 (1.065-17.638)	0.041

BCLC stage					<0.001				<0.001
0	20	6	NA	1		2	NA	1	
A	143	74	51	2.050 (2.892-4.711)	0.091	48	NA	4.119 (1.001-16.951)	0.05
B	22	15	26	4.019 (1.550-10.421)	0.004	12	46	8.992 (2.005-40.320)	0.004
C	27	21	8	6.163 (2.477-15.333)	<0.001	20	13	18.993 (4.419-81.632)	<0.001
Serum AFP									
≤300ng/ml	115	62	48	1		39	NA	1	
＞300ng/ml	94	54	35	1.200 (0.833-1.728)	0.328	43	NA	1.546 (1.002-2.385)	0.049
Miss[b]	3								

Notes: &Information of tumor size was unavailable in 1 patient; φ Information of serum AFP was unavailable in 3 patients. HBV, hepatitis B virus; HCC, hepatocellular carcinoma; BCLC, Barcelona Clinic Liver Cancer; AFP, α-fetoprotein; MRT, median recurrence time; MST, median survival time; RFS, recurrence-free survival; OS, overall survival; HR, hazard ratio; CI, confidence interval; NA, not available; a: one patient data missing of tumor size; b: three patients data missing of serum AFP.
